# Pig Liver Esterase Hydrolysis of 2-Arachidonoglycerol Exacerbates PRRSV-Induced Inflammation via PI3K-Akt-NF-κB Pathway

**DOI:** 10.3390/cells14161227

**Published:** 2025-08-08

**Authors:** Yuelin Fu, Huiwen Zhu, Qiling Xiao, Qi Chen, Qiongqiong Zhou, Xiliang Wang, Deshi Shi

**Affiliations:** 1 State Key Laboratory of Agriculture Microbiology, College of Veterinary Medicine, Huazhong Agricultural University, Wuhan 430070, China; fu123@webmail.hzau.edu.cn (Y.F.); zhw032299@163.com (H.Z.); xiaoqilingk@sina.com (Q.X.); dokic@foxmail.com (Q.C.); zhouqq@cau.edu.cn (Q.Z.); wxl070@mail.hzau.edu.cn (X.W.); 2 Key Laboratory of Development of Veterinary Diagnostic Products, Ministry of Agriculture and Rural Affairs, Wuhan 430070, China

**Keywords:** pig liver esterase, porcine alveolar macrophages, inflammation, Porcine Reproductive and Respiratory Syndrome Virus, 2-arachidonoglycerol

## Abstract

Inflammation is essential for host defense but requires strict regulation to prevent immunopathology. This study reveals how pig liver esterase (PLE) in alveolar macrophages (PAMs) modulates PRRSV-induced inflammation through endocannabinoid metabolism. We identified PLE6 as the dominant hydrolytically active subtype in PAMs. Functional studies demonstrated that PLE promotes pro-inflammatory cytokine expression during PRRSV infection, while its substrate 2-arachidonoylglycerol (2-AG) exerts anti-inflammatory effects. Animal experiments confirmed that PLE inhibition reduces pulmonary inflammation and tissue damage in PRRSV-infected piglets. Transcriptomic and mechanistic analyses revealed that PLE hydrolyzes 2-AG to activate the PI3K-Akt-NF-κB pathway, particularly through enhanced phosphorylation of Akt and p65. These findings establish a novel pathological mechanism where PLE-mediated 2-AG degradation disrupts endocannabinoid homeostasis, amplifying PRRSV-induced inflammation. The study provides therapeutic insights for targeting endocannabinoid hydrolysis to control inflammatory diseases.

## 1. Introduction

Endogenous cannabinoids (eCBs), a class of lipid-based signaling molecules naturally present in mammalian systems, have emerged as a focal point in pharmacological and immunological research due to their extensive regulatory functions. Substantial evidence has established that the anti-inflammatory cascade triggered by eCBs through their interaction with cannabinoid receptors, such as CB2, constitutes a fundamental mechanism in maintaining inflammatory homeostasis within the organism [1,2]. 2-arachidonoylglycerol (2-AG) is a major eCB with significant anti-inflammatory effects. For example, 2-AG can inhibit TLR4-induced immune responses, thereby suppressing the reduction of IL-6, TNF-α, and COX2 expression in macrophages [3]; 2-AG inhibits the virulence program of pathogens by antagonizing the bacterial receptor QseC, thereby reducing the intestinal bacterial load in mice and alleviating colitis [4]. Therefore, 2-AG has attracted increasing attention for its important role in regulating immune and inflammatory responses [5,6]. However, some studies have also found that 2-AG upregulates inflammation after being hydrolyzed by its classic degrading enzyme monoacylglycerol lipase (MAGL), which is a serine hydrolase [7,8,9]. Therefore, inhibiting the activity of 2-AG degrading enzyme can significantly suppress inflammation and is currently one of the popular research targets for inflammation regulation [10,11]. However, the anti-inflammatory mechanisms of 2-AG remains largely confined to neuroscience, metabolic disorders (particularly obesity), cardiovascular protection, and mental health research [12,13,14,15]. Notably, the potential role of 2-AG in modulating viral infection-induced inflammation remains poorly explored, with no existing reports specifically addressing its immunoregulatory function in animal models of viral diseases.

Pig carboxylesterase, as a member of the serine hydrolase family [16], is also known as pig liver esterase (PLE) due to its highest expression level in the liver. The distinctive chiral hydrolytic properties of PLE have established its indispensable role in organic chemical synthesis, consequently directing research efforts over the past century predominantly toward its synthetic applications [17,18,19]. However, the pharmacological significance and physiological functions of PLE in biological systems, particularly its involvement in drug metabolism, nutrient processing, and cellular signaling pathways, remain largely unexplored. Previous studies have demonstrated that mouse carboxylesterase exhibits 2-AG hydrolytic activity surpassing that of MAGL [20]. Similarly, human carboxylesterase has been shown to possess comparable 2-AG hydrolytic capacity to MAGL [21]. PLE and MAGL belong to the serine hydrolase family, and 2-AG contains amide and ester bonds that can be hydrolyzed by PLE [22], therefore, our research team investigated the pharmacological properties of PLE, and discovered that PLE demonstrates superior 2-AG hydrolytic activity compared to MAGL, and that it further promotes LPS-induced inflammatory responses after hydrolyzing 2-AG [23]. This indicates that PLE is closely related to the occurrence and development of inflammation within the organism, potentially serving as a key regulatory node in inflammatory pathways.

Recent studies have revealed significant differences in Porcine reproductive and respiratory syndrome (PRRS) resistance between Tongcheng pigs (Chinese indigenous) and Large White pigs, with Tongcheng pigs showing lower susceptibility to HP-PRRSV [24]. Following experimental HP-PRRSV infection, while both Tongcheng and Large White pigs developed characteristic pneumonia symptoms, Large White pigs exhibited typical pneumonia symptoms after infection, with more severe viral load, lung lesions, and clinical symptoms compared to Tongcheng pigs. Notably, IL-1β and IL-6 were significantly increased in Large White pigs, which may be an important cause of acute lung injury during later infection stages [25,26]. PRRSV, a highly immunosuppressive pathogen, induces severe pulmonary inflammation and tissue damage through its tropism for porcine alveolar macrophages (PAMs)—the primary viral target cells [27,28,29]. This immunoescape strategy frequently results in secondary infections that significantly contribute to disease mortality [30]. Despite extensive research investigating PRRSV-induced pneumonia through multiple approaches—including viral structural and non-structural protein analysis, comparative studies of different strains, and differences in inflammatory response among pigs with different resistance [31,32,33,34]—the mechanisms remain largely elusive, with inconsistent findings and numerous unresolved aspects in the current understanding of this complex pathogenesis. Our previous studies identified distinct PLE expression patterns between these breeds; Tongcheng pigs with mild HP-PRRSV-induced pneumonia displayed lower PLE expression, whereas Large White pigs with severe pneumonia exhibited higher PLE expression [35]. These findings strongly suggest that PLE is likely closely related to PRRSV-induced pneumonia and plays an important regulatory role in its occurrence and development.

Based on the high activity of PLE in hydrolyzing 2-AG, a crucial anti-inflammatory mediator, and inspired by the differential inflammatory responses following HP-PRRSV infection and PLE expression patterns between Tongcheng pigs and Large White pigs, this study systematically investigated the functional role and mechanisms of PLE in PRRSV-induced pneumonia. It is found that inhibiting PLE activity or expression can reduce the inflammation after PRRSV infection, whether in vivo or in vitro, indicating that PLE plays a pro-inflammatory role in PRRSV-induced inflammation. Moreover, this pro-inflammatory effect is achieved through the hydrolysis of endogenous cannabinoid 2-AG. This study reveals the role and mechanism of PLE in PRRSV-induced pneumonia, opening a new window for the pathological understanding of PRRSV, provides a novel theoretical basis for developing PRRS prevention and control strategies, and provides reference for PLE as a promising therapeutic target for inflammatory diseases.

## 2. Materials and Methods

### 2.1. Cloning and Subtype Identification of PLE

Universal PCR primers for PLE (PLE-F and PLE-R; Table S1) were designed based on γPLE cDNA (GenBank: X63323). Total RNA extracted from PAMs using TRIzol (Accurate, Changsha, China) was reverse-transcribed using Evo M-MLV Plus cDNA Synthesis Kit (Accurate, Changsha, China). PCR amplification was performed (94 °C 5 min; 30 cycles of 94 °C 1 min, 57 °C 1 min, 72 °C 2 min; final extension at 72 °C 10 min), followed by 0.8% agarose gel electrophoresis and gel extraction (Omega, Norcross, GA, USA). Purified PLE fragments were ligated into pMD20-T vector (Takara, Beijing, China) at 16 °C overnight and transformed into DH5α competent cells (Tsingke Biotech, Wuhan, China). Positive clones were screened by colony PCR and sequenced (Sangon Biotech, Shanghai China) using M13 F(−47), M13 R(−48), and PLE-M primers (Table S1). Sequences were assembled (DNASTAR), aligned with γPLE (X63323) via NCBI Blast, and translated into amino acid sequences (MEGA) for analysis of twenty-five variant sites across five variable regions [16,35].

### 2.2. Construction of PLE 6 Prokaryotic Functional Expression Plasmid

To amplify the PAMs PLE fragment, PCR was performed using the primer (F:5′-GGAATTCCCATATGGGGGCAGCCAGCCTCGCC-3′, R:5′-CGGCTCCGAGTCCACTTTATCTTGGGTGGCT-3′). The PCR product was then analyzed by 0.8% agarose gel electrophoresis, and the target DNA fragment was excised and purified using the E.Z.N.A.^®^ Gel Extraction Kit (OMEGA, GA, USA) according to the manufacturer’s instructions. The purified PCR product was digested with NdeI and XhoI (Thermo, Waltham, MA, USA) and subsequently ligated into the similarly digested pET15b expression vector. The restriction digestion was carried out at 37 °C for 1 h, followed by overnight ligation at 16 °C using T4 DNA ligase (Thermo, MA, USA). The recombinant plasmids were extracted using the E.Z.N.A.^®^ Plasmid DNA Mini Kit I (OMEGA, GA, USA) following the manufacturer’s protocol. The extracted plasmids were then verified by double digestion with NdeI and XhoI restriction enzymes (Thermo, MA, USA). The successfully digested plasmids were designated as pET15b-PLE6, confirming the correct insertion of the target fragment. The recombinant expression vector pET15b-PLE6 was transformed into Origami-pGro7 competent cells. The transformed cells were plated on LB agar plates supplemented with dual antibiotics (100 µg/mL ampicillin and 25 μg/mL chloramphenicol, Solarbio, Beijing, China) and incubated at 37 °C for 16 h. Single colonies were then selected for bacterial colony PCR verification. Positive clones containing the correct construct were designated as Origami-pGro7-pET15b-PLE6.

### 2.3. Prokaryotic Functional Expression and Purification of PLE6

The Origami-pGro7-pET15b-PLE6 transformants were inoculated into LB medium containing both ampicillin (100 μg/mL) and chloramphenicol (25 μg/mL). The culture was supplemented with 1 mg/mL L-arabinose (Solarbio, Beijing, China) at initiation and grown at 30 °C with shaking at 200 rpm. When the OD600 reached 0.6–0.8 (after 2–3 h of cultivation), PLE6 expression was induced by adding isopropyl β-D-1-thiogalactopyranoside (IPTG, Solarbio, Beijing, China) to a final concentration of 40 μM. Following 6 h of induction, cells were harvested by centrifugation at 8000× *g* for 10 min at 4 °C. The bacterial cells were lysed using a low-temperature ultra-high pressure continuous flow cell disruptor (JN-02C, Guangzhou, China) until the effluent became clear. The lysate was then centrifuged at 12,000× *g* for 30 min at 4 °C to collect the supernatant. Following clarification through a 0.45 μm filter membrane (Millipore, Burlington, MA, USA), the supernatant was subjected to affinity chromatography purification using an AKTApurifier system (Cytiva, Uppsala, Sweden) equipped with a HisTrap FF column (Cytiva, Uppsala, Sweden). The purified protein fractions were analyzed by SDS-PAGE, and the target-containing fractions were pooled and concentrated to approximately 1 mL using an ultrafiltration tube (Millipore, MA, USA) at 4000× *g* and 4 °C. The final protein product was aliquoted and stored at –80 °C for subsequent use.

### 2.4. Identification of PLE 6 Hydrolysis Activity

The enzymatic reaction was performed in a 1 mL reaction system at room temperature using 50 mM PBS buffer (pH 7.2). The reaction mixture contained 1 mM p-nitrophenyl acetate (p-NPA, Sigma-Aldrich, St. Louis, MO, USA) as substrate and 10 μg of prokaryotically expressed PLE6. P-NPA is a universal substrate for carboxylesterase, which is hydrolyzed to produce p-NP with a yellow solution.

### 2.5. Cell Culture and Virus Propagation

Porcine alveolar macrophages (PAMs) were obtained from the postmortem lung lavage of 4-week-old specific-pathogen-free (SPF) pigs and maintained in an RPMI medium 1640 supplemented with 10% heat-inactivated FBS (Gibco, Carlsbad, CA, USA). Marc-145 cells were cultured in a Dulbecco modified Eagle medium (DMEM) with 10% FBS. The PRRSV (WUH3, GenBank: HM853673.1) was propagated on PAMs or Marc-145 cells, and the virus titers were determined. Briefly, PRRSV was serially diluted 10-fold in complete DMEM or RPMI 1640 to infect 5 × 10^4^ Marc-145 cells or PAMs in 96-well plates. Virus titer was determined using the Reed-Muench method and expressed as the TCID50. The virus was stored at −80 °C until use.

### 2.6. PLE Activity Inhibition and Virus Infection

PAMs were pre-treated with 100 μM BNPP (Sigma, MO, USA), a specific PLE inhibitor, for 3 h prior to the PRRSV strains’ WUH3 infection (MOI = 0.5). Subsequently, the medium was replaced with a fresh DMEM maintenance medium containing 2% FBS. This was maintained until 6, 12, or 24 h post-infection (hpi) for sample collection. Total RNA was extracted for RT-qPCR analysis of IL-1β, IL-6, and TNF-α expression.

### 2.7. RNA Extraction and Real-Time PCR

According to the manufacturer’s instructions, total RNA was extracted from treated cells using TRIzol (Accurate, Changsha, China). HiScript ^®^ II Q RT SuperMix (Vazyme, Nanjing, China) was used to prepare cDNA from RNA. Real-time fluorescence quantitative PCR analysis was performed using a real-time fluorescence quantitative PCR system (BIO-RAD, Hercules, CA, USA) and ChamQ Blue Universal SYBR qPCR Master Mix (Vazyme, Nanjing, China) real-time fluorescence quantitative PCR premix. The primers used for IL-1β, IL-6, IL-8, IL-17, TNF-α, PI3K, Akt, NF-κB, and GAPDH are shown in Table S2.

### 2.8. Western Blot Analysis

Total protein was extracted from cells using a RIPA lysis buffer (CWBIO, Taizhou, China) containing phosphatase and protease inhibitor (CWBIO, Taizhou, China). Protein concentration was quantified using the BCA kit (Beyotime, Shanghai, China). The protein sample (15 μg) was separated on 10% SDS-PAGE gel and transferred to a PVDF membrane (Millipore, MA, USA). Membranes were blocked with 8% skim milk or 5% BSA at 4 °C for 6–8 h, and incubated overnight at 4 °C with primary antibody: anti-PI3K (1:10,000, Proteintech, Wuhan, China), anti-p-Akt (1:2000, Vazyme, Nanjing, China), anti-Akt (1:10,000, Proteintech, Wuhan, China), anti-p-p65 (1:5000, Proteintech, Wuhan, China), anti-p65 (1:2000, Proteintech, Wuhan, China), anti-PLE (1:500, Prepared by our team), and anti-GAPDH (1:4000. Servicebio, Wuhan, China). Then, the membrane was incubated with appropriate secondary antibodies (1:10,000, CWBIO&Biodragon, Taizhou&Beijing, China) at room temperature for 1 h. The membranes were imaged by first combining Liquid A and Liquid B from the enhanced chemiluminescence detection kit (Epizyme, Shanghai, China) to configure the corresponding volume of the substrate working solution at a 1:1 ratio. The solution was evenly dropped onto the imprinting membrane to image the membrane, and the Chemi DocTMXRS+ (BIO-RAD, CA, USA) was used for imaging.

### 2.9. siRNA Knockdown

According to the manufacturer’s instructions, GP-transfect Mate transfection reagent (GenePharma, Suzhou, China) was used to transfect siRNA targeting PLE or non-specific control (NC). siRNA was synthesized by GenePharma, and the sequence is shown in Table S3. We transfect PAMs with 50 nM siRNA, harvest the cells 24 h later, and evaluate the efficiency of PLE silencing by RT- qPCR and WB.

### 2.10. PLE Enzyme Activity Assay and 2-AG Hydrolysis

An amount of 50 μg of PAMs lysate was incubated with the carboxylesterase universal substrate p-NPA (1 mmol/L, Sigma, MO, USA) in PBS buffer (50 mmol/L, pH 7.2) at room temperature. The hydrolysis product p-NP has a maximum absorbance at 405 nm. We performed hydrolysis of 2-AG (Cayman, Ann Arbor, MI, USA) using the prokaryotic functional expression of PLE6 with PBS (50 mmol/L, pH 7.2) as the hydrolysis buffer. Final concentrations of 2-AG were at 1, 5, and 10 μmol/L, the PLE6 dosage at 5 μg, the total reaction volume at 100 μL; the hydrolysis reaction temperature was at 37 °C for 30 min, then filtered through a 0.22 μm filter and added to PAMs culture medium.

### 2.11. RNA-Seq and Data Analysis

We extracted PAMs RNA from the animal experiments using TRIzol (Invitrogen, Carlsbad, CA, USA). RNA integrity was assessed using the Bioanalyzer 2100 system (Agilent Technologies, Santa Clara, CA, USA), and the data were confirmed by denatured agarose gel electrophoresis. After library quality control, different libraries were pooled based on the effective concentration and targeted data amount. The 5′-end of each library was phosphorylated and cyclized. Subsequently, loop amplification was performed to generate DNA nanoballs. These DNA nanoballs were finally loaded into flowcell with DNBSEQ-T7(MGI, Shenzhen, China) for sequencing. The library construction, quality inspection, and machine sequencing were all completed by Novogene Technology Co., Ltd. in Beijing. We calculated the padj-value and fold change (FC) of each gene, screened differentially expressed genes (DEGs) with padj < 0.05 and |FC| > 1.5, and then performed gene ontology function (GO) and Kyoto Encyclopedia of Genes and Genomes (KEGG) pathway enrichment analysis on DEGs.

### 2.12. ELISA

According to the instructions, use the ELISA kit (HYCEZMBIO, Wuhan, China) to measure the concentration of IL-1β, IL-8, IL-17, AA, PGD2, and PGE2 in the serum.

### 2.13. Animal Experiment

Twelve one month old SPF piglets (obtained from Wuhan Golden Dragon Group) were randomly divided into three groups. Four piglets in the control group were treated with physiological saline solution. Four piglets in each of the infection group and the PLE inhibitor BNPP (Sigma, MO, USA) group were infected with PRRSV (WUH3 10^5^ TCID_50_/mL) by intranasal inoculation of 1 mL and intramuscular injection of 2 mL. After the appearance of clinical symptoms, four piglets were treated with BNPP (25 mg/kg, intraperitoneal injection) [23]. After 24 h of BNPP injection, all piglets were anesthetized via intravenous administration of Propofol (3 mg/kg, jiabopharm, Qingyuan, China). Following confirmation of successful anesthesia (as evidenced by loss of palpebral reflex and muscle tone), euthanasia was performed by exsanguination and samples were collected.

### 2.14. Immunofluorescence

The fixed cells were treated with 4% paraformaldehyde for 10 min at 4 °C, washed three times with ice-cold PBS (5 min each), then permeabilized with 0.2% Triton X-100 (Beyotime, Shanghai, China) for 10 min. After blocking with PBST (PBS containing 0.1% Tween-20) supplemented with 1% BSA and 22.5 mg/mL glycine for 30 min, samples were incubated with anti-p65 primary antibody (1:100; Proteintech, Wuhan, China) overnight at 4 °C, followed by incubation with Cy3 conjugated Goat Anti-mouse IgG (H + L) secondary antibody (1:200; Servicebio, Wuhan, China) for 1 h at room temperature in the dark. Nuclei were counterstained with DAPI (Servicebio, Wuhan, China) for 5 min before fluorescence imaging using a Zeiss Axio Observer Z1 microscope (Oberkochen, Germany).

### 2.15. Statistical Analysis

Statistical analysis was performed using GraphPad Prism 8.0 software, and differences were analyzed using Student’s *t* test. Significance is denoted in the figures as follows: *, *p* < 0.05; **, *p* < 0.01; ***, *p* < 0.001.

## 3. Results

### 3.1. Characterization of PLE Expression in PAMs and Its Functional Involvement in PRRSV Infection

**Figure 1:**
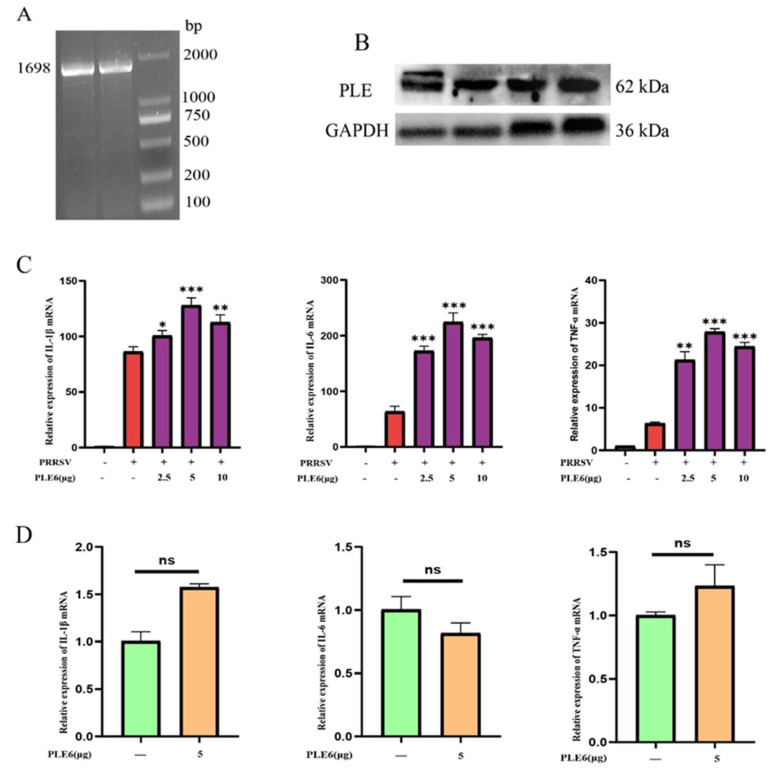
Characterization of PLE expression in PAMs and its functional involvement in PRRSV infection. (**A**) RT-PCR amplification of PLE ORF in 1-month-old piglet PAMs. (**B**) Western Blot analysis of PLE expression in 1-month-old piglet PAMs. (**C**) RT-qPCR analysis of prokaryotic functional expression PLE6 effects on IL-1β, IL-6, and TNF-α expression in PRRSV-infected PAMs. (**D**) The addition of prokaryotically expressed PLE6 alone was analyzed for its impact on the expression of IL-1β, IL-6, and TNF-α through RT-qPCR in PAMs. The data are representative of three independent experiments (means the standard errors of the mean [SEM]). *, *p* < 0.05; **, *p* < 0.01; ***, *p* < 0.001 (Student’s *t* test).

Verification of PLE expression and its abundance in PAMs constituted the fundamental basis of this study. We therefore initially investigated the expression profile of PLE in PAMs. To characterize PLE expression in PAMs, the ORF of PAMs PLE was amplified by PT-RCR. Agarose gel electrophoresis analysis of the amplification products revealed a distinct 1698 bp band (Figure 1A), corresponding to the expected size of the PLE transcript. Subsequent western blot analysis confirmed substantial PLE protein expression in PAMs (Figure 1B), demonstrating high endogenous levels of this enzyme in PAMs. Given that PLE constitutes an enzyme family comprising multiple isoenzymes with varying hydrolytic capacities across different subtypes [23,36], further research has been conducted on the types of PLE subtypes within PAMs. Following the classification method established by Xiao and Brusehaber et al. [16,35], our analysis of 75 positive clones revealed that 69 clones (92%) encoded PLE6, while other subtypes accounted for only 8% (Table 1; detailed PLE subtype amino acid sequences are provided in Additional files). These findings fully demonstrate that PLE has a high expression in PAMs, and PLE 6 is the main subtype.

Following the identification of PLE6 as the predominant subtype in PAMs, we investigated its functional role in PRRSV-induced inflammation. Prokaryotic functional expression of the main subtype PLE6 was performed and added to PAMs infected with PRRSV. It was observed that PLE6 significantly increased the expression of pro-inflammatory factors in PRRSV-infected PAMs (Figure 1C), and the addition of PLE6 alone did not have a pro-inflammatory effect (Figure 1D). This indicates that PLE plays a pro-inflammatory role in PRRSV infection, but PLE itself does not have pro-inflammatory function, and this pro-inflammatory effect depends on other mediators or pathways to achieve.

**Table 1:** Cloning statistics of PLE subtypes in PAMs.

Subtype	Number	Ratio
PLE6	69	92%
Others	6	8%

### 3.2. PLE Inhibition and Knockdown Suppress PRRSV-Mediated Inflammation

**Figure 2:**
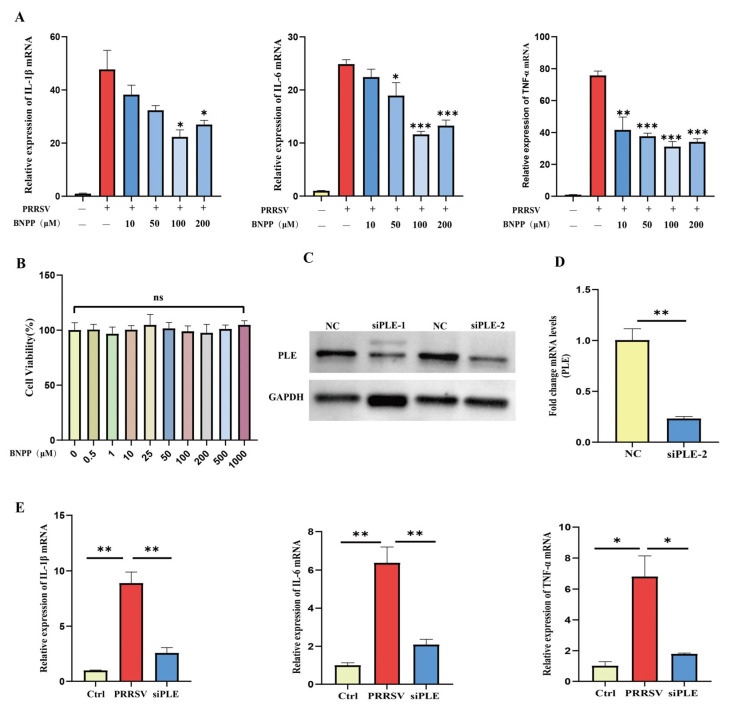
PLE inhibition and knockdown suppressed PRRSV-mediated inflammation. (**A**) RT-qPCR analysis of IL-1β, IL-6, and TNF-α expression in PRRSV-infected PAMs pre-treated with BNPP. (**B**) The effect of different concentrations of BNPP treatment on cell viability. (**C**,**D**) PLE knockdown efficiency was verified by western blot analysis and RT-qPCR. (**E**) The effect of PLE knockdown on the expression of IL-1β, IL-6, and TNF-α in PRRSV infected PAMs. The data are representative of three independent experiments (means the standard errors of the mean [SEM]). *, *p* < 0.05; **, *p* < 0.01; ***, *p* < 0.001 (Student’s *t* test).

Subsequently, we modulated PLE activity and expression to further elucidate its role in PRRSV-induced inflammation in PAMs. Firstly, the specific inhibitor Bis (4-nitrophenyl) phosphoric acid (BNPP) of PLE was used to inhibit its activity prior to infection. It was observed that BNPP demonstrated no detectable cytotoxicity at from 0.5 μM to 1000 μM (Figure 2A) and BNPP significantly downregulated the expression of IL-1β and IL-6 induced by PRRSV, and the expression of TNF-α also decreased (Figure 2B). Next, to further investigate the role of PLE in the expression of pro-inflammatory cytokines induced by PRRSV, PLE expression was knocked down by targeted siRNA transfection (Figure 2C,D). Silencing PLE resulted in marked reduction of PRRSV-induced IL-1β and TNF-α expression, along with decreased IL-6 production (Figure 2E). These consistent findings indicate that inhibiting PLE activity and expression can suppress the expression of pro-inflammatory factors after PRRSV infection, suggesting that PLE plays a pro-inflammatory role in HP-PRRSV infection.

### 3.3. 2-AG Inhibits PRRSV-Induced Inflammation but Promotes It Post-Hydrolysis

**Figure 3:**
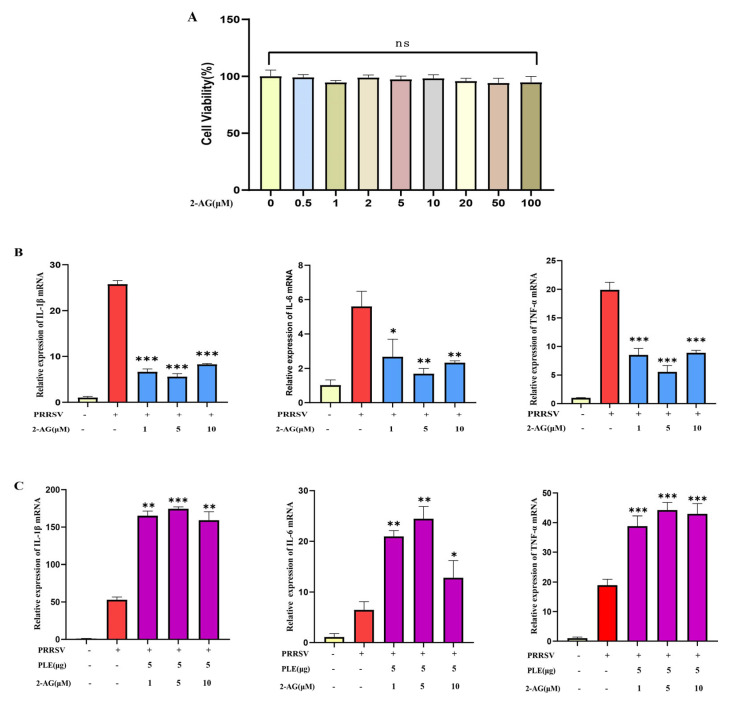
2-AG inhibited PRRSV-induced inflammation but promoted it post-hydrolysis. (**A**) The effect of different concentrations of 2-AG on cell viability. (**B**) The effect of 2-AG (1 μM, 5 μM, or 10 μM) on the expression of IL-1β, IL-6, TNF-α in PRRSV infected PAMs was analyzed by RT-qPCR. (**C**) The effect of 2-AG (1 μM, 5 μM, or 10 μM) hydrolysis on the expression of IL-1β, IL-6, TNF-α in PRRSV infected PAMs was analyzed by RT-qPCR. The data are representative of three independent experiments (means the standard errors of the mean [SEM]). *, *p* < 0.05; **, *p* < 0.01; ***, *p* < 0.001 (Student’s *t* test).

Although the anti-inflammatory properties of 2-AG were well-documented, it remains unknown if 2-AG plays the same role in PRRSV infection. In this study, we investigated the role of 2-AG in PRRSV-induced inflammatory response. Different concentrations of 2-AG were used to treat PAMs infected with PRRSV; 0.5–100 μM of 2-AG revealed no detectable cytotoxicity (Figure 3A) and 1–10 μmol of 2-AG significantly inhibited the expression of IL-1β, IL-6, and TNF-α induced by PRRSV (Figure 3B). The results indicated that 2-AG could inhibit the expression of PRRSVP-induced pro-inflammatory cytokines. However, when 2-AG was pre-hydrolyzed by PLE and then added to PRRSV-infected PAMs, this anti-inflammatory effect was abolished, accompanied by a significant increase in pro-inflammatory cytokine expression (Figure 3C). This demonstrates that the hydrolytic products of 2-AG exhibit pro-inflammatory effects, and that PLE-mediated hydrolysis of 2-AG serves as the crucial mechanism underlying its pro-inflammatory action.

### 3.4. Inhibition of PLE Activity Reduces Lung Inflammation and Tissue Damage in PRRSV-Infected Pigs

**Figure 4:**
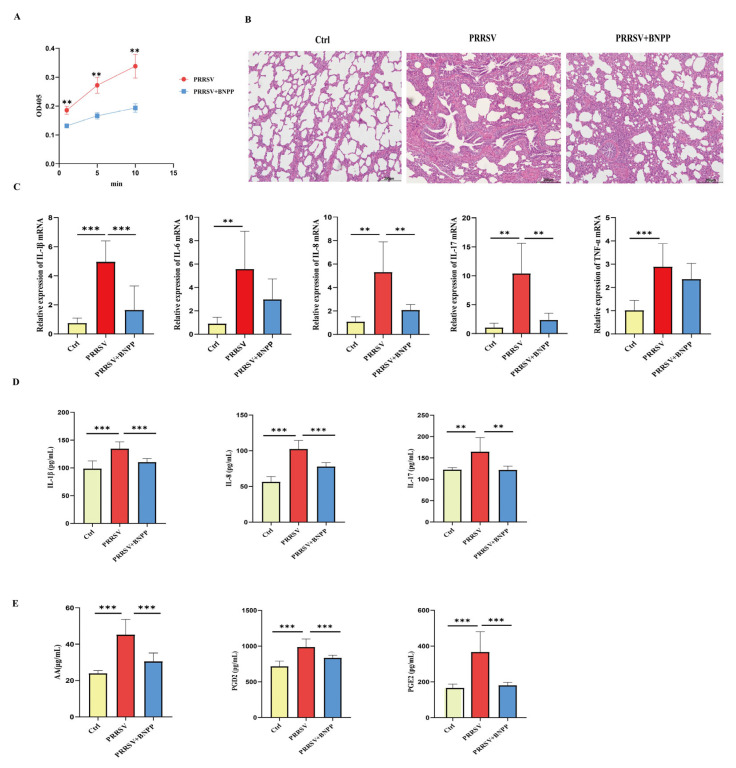
PLE inhibition reduced the degradation of 2-AG and weakened pulmonary inflammation in PRRSV infected pigs. (**A**) PLE enzymatic activity in PAM lysates was determined using p-NPA hydrolysis assay. (**B**) Pulmonary histopathology was assessed by H&E staining. (**C**) Expression of pro-inflammatory cytokines (IL-1β, IL-6, IL-8, IL-17, TNF-α) in PAMs were analyzed by RT-qPCR. (**D**) Serum concentration of IL-1β, IL-8, and IL-17 were quantified by ELISA. (**E**) Serum concentration of AA, PGD2, and PGE2 were analyzed by ELISA. Data are presented as the means ± SEM. **, *p* < 0.01; ***, *p* < 0.001 (Student’s *t* test).

Building upon these findings, we further investigated PLE’s regulatory role in PRRSV-induced inflammation at the organismal aspect. Following experimental PRRSV infection, all eight piglets developed characteristic clinical symptoms including pyrexia, lethargy, and coughing. At the onset of clinical signs, four piglets received intraperitoneal administration of the PLE-specific inhibitor BNPP (25 mg/kg). Twenty-four hours post-treatment, all animals were euthanized for sample collection. Firstly, the PLE enzyme activity of the isolated PAMs was rapidly measured, and accomparative assessment of PLE enzymatic activity in PAM lysates indicated that BNPP treatment substantially reduced p-NPA hydrolysis, providing direct evidence of pharmacological inhibition of PLE activity in vivo (Figure 4A).

Subsequently, we evaluated the protective effects of PLE inhibition against PRRSV-induced pulmonary pathology. Histopathological analysis revealed severe interstitial pneumonia in infected piglets, characterized by alveolar septal thickening, epithelial hyperplasia, and marked alveolar collapse, along with bronchial epithelial desquamation and inflammatory cell exudation. Importantly, BNPP-mediated PLE inhibition significantly attenuated these pathological manifestations (Figure 4B). Analysis of pro-inflammatory factors in both PAMs and serum revealed that PRRSV infection markedly upregulated key inflammatory cytokines, including IL-1β, IL-8, and IL-17, while BNPP inhibition of PLE activity significantly downregulated the expression of these pro-inflammatory factors (Figure 4C,D).

Furthermore, metabolic profiling of 2-AG in serum demonstrated that BNPP treatment substantially reduced concentration of arachidonic acid (AA), the primary 2-AG metabolite, along with its downstream eicosanoids PGE2 and PGD2, compared to HP-PRRSV-infected controls (Figure 4E). These comprehensive findings demonstrate that inhibiting PLE activity in vivo weakens the degradation of 2-AG by PLE and significantly reduces the inflammatory level in piglets.

### 3.5. The PI3K-Akt-NF-κB Pathway Is Important for Reducing Inflammation After PRRSV Infection Through PLE Inhibition

**Figure 5:**
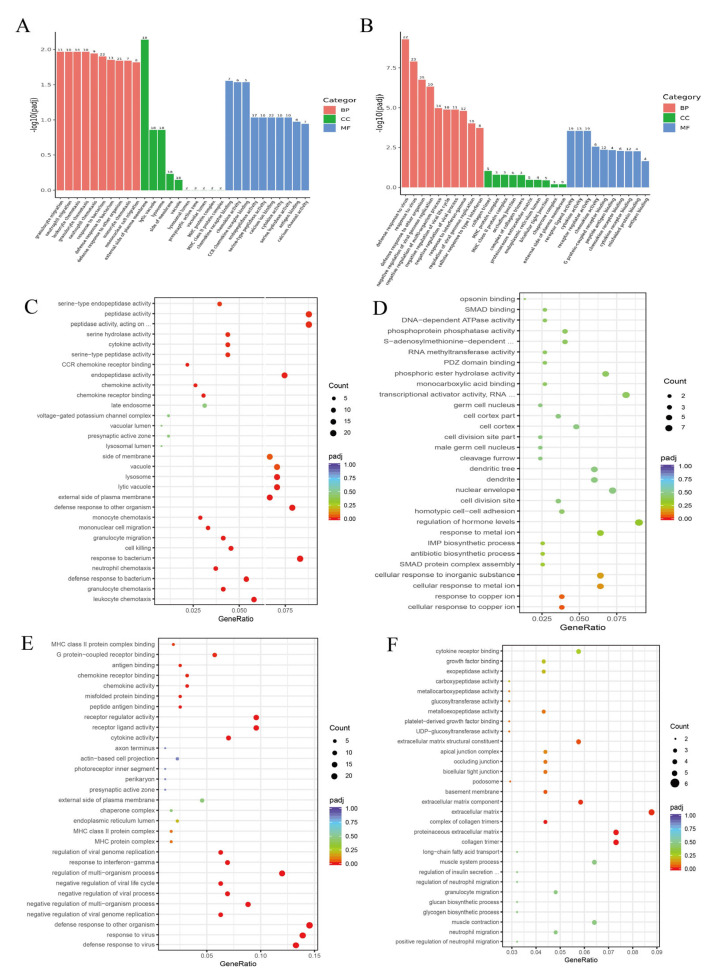
RNA-seq Gene Ontology (GO) Enrichment. (**A**) PRRSV/Ctrl group GO all Term. (**B**) BNPP/PRRSV group GO all Term. (**C**) PRRSV/Ctrl group GO up Term. (**D**) PRRSV/Ctrl group GO down Term. (**E**) BNPP/PRRSV group GO up Term. (**F**) BNPP/PRRSV group GO down Term. ... in (**C**): L-amino acid peptides; top ... in (**D**) methyltransferase activity; bottom ... in (**D**): polymerase II transcription regulatory region sequence-specific DNA binding; ... in (**F**): involved in cellular response to glucose stimulus.

To elucidate the molecular mechanisms underlying PLE-mediated regulation of inflammatory responses in PRRSV-infected piglets, we performed transcriptomic sequencing of PAMs from the piglets. Differential gene expression analysis was conducted using a threshold of padj < 0.05 and |Fold Change| > 1.5, followed by GO and KEGG enrichment analyses of identified differentially expressed genes. The data demonstrated that in the GO enrichment of the PRRSV/Ctrl group, the main enriched terms were granulocyte migration, monocyte migration, defense response to bacterium, chemokine activity, serine hydrolase activity, calcium channel activity, etc. (Figure 5A), among which the upregulated main terms were granulocyte chemotaxis, chemokine activity, serine hydrolase activity, etc. (Figure 5C). The main terms downregulated were cell response to copper ions, opsin binding, etc. (Figure 5D). In the GO enrichment results of the BNPP/PRRSV group, the main enriched terms were defense response to virus, negative regulation of virus progression, response to interferon-gamma, cytokine activity, G protein coupled receptor binding, antigen binding, etc. (Figure 5B), among which the upregulated main terms were response to virus, cytokine activity, chemokine activity, etc. (Figure 5E). The main terms downregulated are carboxypeptidase activity, glycogen biosynthesis process, etc. (Figure 5F).

**Figure 6:**
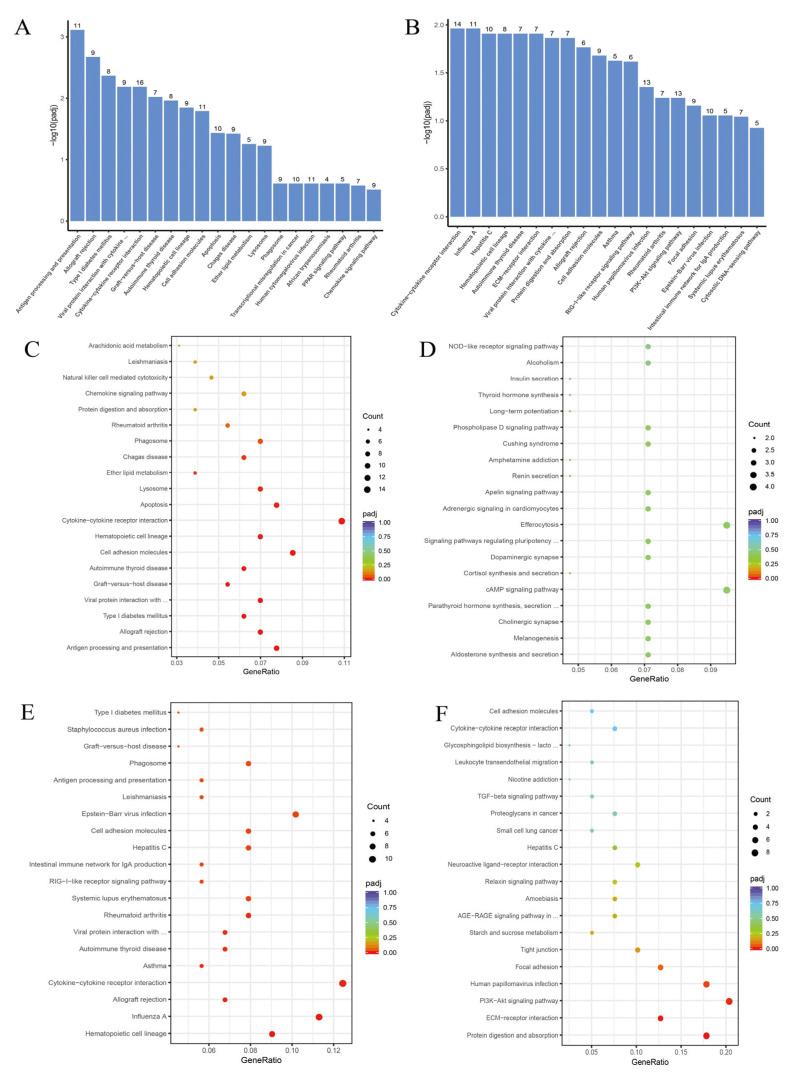
RNA-seq Kyoto Encyclopedia of Genes and Genomes (KEGG) Enrichment. (**A**) PRRSV/Ctrl group KEGG all Term. (**B**) BNPP/PRRSV group KEGG all Term. (**C**) PRRSV/Ctrl group KEGG up Term. (**D**) PRRSV/Ctrl group KEGG down Term. (**E**) BNPP/PRRSV group KEGG up Term. (**F**) FBNPP/PRRSV group KEGG down Term. ... in (**A**): and cytokine receptor; ... in (**B**): and cytokine receptor; ... in (**C**): cytokine and cytokine receptor; top ... in (**D**): of stem cells; bottom ... in (**D**): and action; ... in (**E**): cytokine and cytokine receptor; top ... in (**F**): and neolacto series; bottom ... in (**F**): diabetic complications.

In the KEGG enrichment results, the PRRSV/Ctrl group mainly enriched terms such as antigen processing and presentation, cell adhesion molecules, apoptosis, arachidonic acid metabolism, ether ester metabolism, etc. (Figure 6A), among which the upregulated terms were mainly ether ester metabolism, chemokine signaling pathway, arachidonic acid metabolism, etc. (Figure 6C), and the downregulated terms enriched were mainly aldosterone secretion synthesis, cortisol secretion synthesis, NOD-like receptor signaling pathway, etc. (Figure 6D). The BNPP/PRRSV group mainly enriched terms such as protein digestion and absorption, cell adhesion molecules, RIG-I like receptor signaling pathway, PI3K Akt signaling pathway, etc. (Figure 6B), among which the upregulated terms were mainly cytokine receptor interactions, virus protein interactions with cytokine, RIG-I like receptor signaling pathway, cell adhesion molecules, etc. (Figure 6E), and the downregulated terms enriched were mainly PI3K Akt signaling pathway, protein digestion and absorption, ECM receptor interactions, focal adhesion, etc. (Figure 6F).

**Figure 7:**
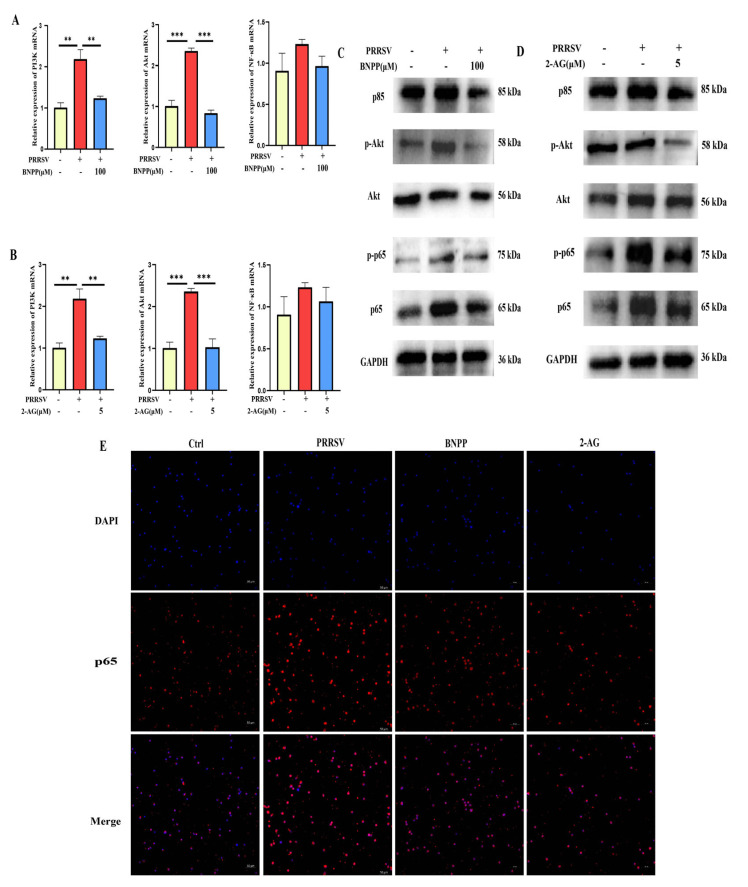
PLE activity inhibition suppressed PRRSV-induced PI3K-AKT-NF-κB pathway activation. (**A**) RT-qPCR analysis of PI3K, Akt, and NF-κB expression in PRRSV infected PAMs pre-treated with BNPP (100 μM). (**B**) RT-qPCR analysis of PI3K, Akt, and NF-κB expression in PRRSV-infected PAMs treated with 2-AG (5 μM). (**C**) Western blot analysis of PI3K, p-Akt, Akt, p-p65, and p65 expression in PRRSV infected PAMs pre-treated with BNPP (100 μM). (**D**) Western blot analysis of PI3K, p-Akt, Akt, p-p65, and p65 expression in PRRSV-infected PAMs treated with 2-AG (5 μM). (**E**) IF analysis of p65 translocation in PRRSV-infected PAMs with BNPP (100 μM)/2-AG (5 μM) treatment. The data are representative of three independent experiments (means the standard errors of the mean [SEM]). **, *p* < 0.01; ***, *p* < 0.001 (Student’s *t* test).

Integrating literature evidence with transcriptomic findings, we identified the PI3K-Akt-NF-κB signaling pathway as a crucial pathway in PLE-mediated modulation of PRRSV-induced inflammation. Subsequent validation in PAMs demonstrated that PRRSV infection markedly upregulated PI3K and Akt mRNA expression, while PLE inhibition significantly attenuated this upregulation and concurrently suppressed NF-κB expression (Figure 7A). The protein level results were basically consistent with the mRNA level. In particular, it was found that inhibiting PLE activity could suppress the phosphorylation degree of Akt and p65 (Figure 7C).

In previous studies, it was found that 2-AG can inhibit the expression of pro-inflammatory cytokines caused by PRRSV infection in PAMs, while PLE can efficiently hydrolyze 2-AG. Therefore, the question of whether the effect of inhibiting PLE activity on the aforementioned pathway is related to 2-AG has been explored. The results showed that 2-AG can significantly downregulate the expression of PI3K and Akt mRNA levels after infection, and also inhibit the expression of NF-κB (Figure 7B). The western blot analysis results were consistent with the mRNA level, and the phosphorylation of Akt and p65 was also inhibited (Figure 7D). Furthermore, the p65 expression and nuclear translocation in PRRSV-infected PAMs following BNPP or 2-AG treatment were assessed by immunofluorescence. PRRSV infection markedly increased both p65 expression and nuclear accumulation, whereas BNPP and 2-AG attenuated this effect (Figure 7E). These observations paralleled the effects of PLE inhibition indicating that indicating that BNPP inhibits the activation of this pathway by suppressing the degradation of 2-AG by PLE.

## 4. Discussion

Previous investigations have established that PLE exhibits its highest expression in hepatic tissue [35], while systematic analyses of its expression in other cell types remain limited. As a multi-isoenzyme family, PLE demonstrates subtype-specific hydrolytic activities. Although the main target cells of PRRSV infection are PAMs in the lung, PLE expression in PAMs has not been characterized. As the principal resident immune cells in alveolar spaces, PAMs serve as both initiators and regulators of inflammatory responses in most cases of pathogen-induced pneumonia. These macrophages play a pivotal role in maintaining pulmonary homeostasis and regulating inflammatory processes [37,38,39]. Therefore, clarifying the expression of PLE in PAMs is of great significance for understanding the regulation of PLE in PRRSV-induced pneumonia.

In this study, we characterized PLE expression and subtype distribution in PAMs isolated from 1-month-old piglets. Our findings revealed substantial PLE expression in PAMs, with sequencing analysis identifying PLE6 as the predominant subtype, accounting for 92% of total PLE expression, while other subtypes collectively represented only 8%. Interestingly, this expression pattern contrasts sharply with our previous findings in hepatic tissue, where we identified up to 42 distinct PLE subtypes, none of which exceeded 50% in relative abundance [35]. The predominant expression of PLE6 in PAMs represents a remarkable tissue-specific distribution pattern which is worth considering. We speculate that PLE potentially exhibits cell-type-specific subtype expression. Hepatic tissue comprises diverse cell populations including hepatocytes, Kupffer cells, adipocytes, and epithelial cells, while PAMs represent a more homogeneous cell population, potentially explaining the observed subtype distribution differences. Our previous investigations revealed that PLE exhibits significant substrate preference for 2-AG hydrolysis with ester bonds (unpublished data). As the predominant innate immune cells in porcine respiratory defense, PAMs play a pivotal role in nonspecific immunity. The main expression of PLE6 in PAMs may suggest an evolutionarily conserved mechanism whereby PLE-mediated 2-AG hydrolysis serves as a crucial regulatory way in macrophage immunomodulation. This likely represents a fundamental pathway through which PLE modulates PAM immune functions. PLE expresses specific subtypes within specific cells, which is also a manifestation of PLE’s need to better adapt to different cells and achieve its own physiological functions.

Furthermore, this distinct expression pattern likely reflects tissue-specific functional adaptations, suggesting that PLE subtype composition is closely associated with the physiological requirements of its expression tissue. Current understanding of PLE’s physiological functions primarily centers on its catalytic role in hydrolyzing both endogenous and exogenous compounds. For instance, PLE mediates the hydrolysis of 1-methyl-1-cyclopropylmethyl derivatives, generating products that serve as potent histone deacetylase inhibitors with potential therapeutic applications in cancer and neurodegenerative disorders. Additionally, PLE demonstrates stereoselective catalytic activity in the hydrolysis of racemic 1,2-dimethoxycarbonyl-4-oxocyclopentane, producing R, R-diesters that exhibit anti-HIV activity [40,41]. These findings comprehensively illustrate the robust hydrolytic capacity of PLE as a critical enzyme that aligns precisely with the liver’s physiological role as the body’s primary metabolic organ. This functional correlation is evidenced by the substantial expression of multiple PLE subtypes in hepatic tissue, facilitating efficient metabolism of various chemicals. In contrast, the pulmonary system, primarily serving respiratory functions, demonstrates limited requirement for extensive hydrolytic and metabolic processing of chemical compounds, so it may not be necessary to express superfluous PLE subtypes. Consequently, the unique expression patterns of PLE may represent a crucial regulatory mechanism through which this enzyme facilitates specialized cellular functions across different tissues. Future investigations should focus on systematically characterizing the expression patterns and relative abundance of PLE subtypes in various tissue types and cells. Such comprehensive analyses will provide valuable insights into the physiological roles and potential pathological implications of PLE in tissue-specific contexts.

In the previous section we systematically analyzed the potential associations among PLE, 2-AG, and PRRSV infection. Our experimental results demonstrated that inhibition of PLE activity and expression markedly attenuated PRRSV-induced pro-inflammatory cytokines. At the same time, it found that 2-AG can directly inhibit the pro-inflammatory cytokines expression induced by PRRSV infection, which has not been reported in previous studies on the anti-inflammatory effect of 2-AG, and this anti-inflammatory effect disappears after hydrolysis and further promotes inflammation. These results preliminarily indicate that PLE plays a pro-inflammatory role in PRRSV infection, and this effect is achieved through hydrolysis of 2-AG.

AA is the primary metabolic product of 2-AG hydrolysis and a crucial omega-6 polyunsaturated fatty acid with significant biological implications [42,43]. It is not only an important substance for maintaining cell membrane structure and function, but also a precursor for various bioactive substances such as prostaglandins, leukotrienes, and thromboxanes. The pro-inflammatory effects of substances such as AA, PGE2, and PGD2 have been extensively studied [44,45]. In our results, inhibition of PLE activity reduced 2-AG degradation, which not only enhanced its anti-inflammatory effects but also decreased the production of pro-inflammatory metabolites. Consequently, the BNPP-treated group exhibited significantly lower inflammatory levels and tissue damage compared to the PRRSV-infected group. These observations were further supported by histopathological analysis and pro-inflammatory cytokine detection. Our findings position PLE as a key pro-inflammatory regulator in PRRSV pathogenesis, and a promising broad-spectrum target for inflammatory disease intervention.

To further investigate the mechanism by which PLE promotes inflammation caused by PRRSV infection, we conducted comprehensive transcriptome sequencing analysis on PAMs isolated from in vivo experiments. Notably, GO enrichment analysis of the PRRSV/Ctrl group revealed significant upregulation of serine hydrolase activity. PLE is indeed a serine hydrolase. Correlation analysis of our experimental data suggests that the enhanced serine hydrolase activity likely reflects upregulated PLE hydrolytic function, which is directly related to high-level inflammation in the PRRSV infection group. At the same time, KEGG pathway enrichment analysis of the PRRSV/Ctrl group revealed significant upregulation of arachidonic acid metabolism, which was also reported in Liang et al.’s study [46]. This suggests enhanced arachidonic acid metabolic activity in piglets following PRRSV infection, which aligns consistently with our in vivo experimental data. These results provide important support for the claim that PLE enhances arachidonic acid metabolism through hydrolysis of 2-AG, leading to an increase in inflammation levels.

Furthermore, upregulation of the “defense response to bacterium” was also enriched in the PRRSV/Ctrl group, indicating that the PRRSV infection group is likely to have secondary bacterial infection. This observation is consistent with previous findings by Thanawongnuwech et al. [47,48]. Secondary bacterial infection is an important cause of severe inflammation. The management of inflammatory responses induced by secondary infections represents a critical challenge that extends far beyond PRRSV control. Numerous clinical and experimental observations have demonstrated that primary viral infections often establish favorable conditions for secondary infections through various mechanisms, leading to exacerbated inflammatory pathology. A well-documented example involves respiratory syncytial virus and rhinovirus infections, which significantly increase susceptibility to secondary pneumococcal infections by compromising host defense mechanisms, ultimately resulting in severe pulmonary inflammation [49]; pseudorabies virus infection enhances respiratory barrier permeability and consequently promotes superinfection with *Pasteurella multocida*, leading to aggravated tissue damage [50]. This compellingly demonstrates that controlling secondary infections and their consequent inflammatory damage represents both a scientifically significant and clinically challenging imperative. In addition, the endocannabinoid metabolic homeostasis plays a critical role in modulating systemic inflammatory responses. Beyond the well-characterized endocannabinoid-degrading enzymes, other hydrolytic enzymes—including serine hydrolases such as PLE—may disrupt endocannabinoid balance by interfering with the endocannabinoid system, consequently influencing inflammatory regulation. Therefore, inhibiting their hydrolysis of endocannabinoids to suppress excessive inflammation is a potential strategy for controlling inflammatory diseases.

In the KEGG pathway enrichment analysis of the BNPP/PRRSV group, we identified significant downregulation of the PI3K-Akt signaling pathway, a crucial intracellular signaling cascade comprising phosphatidylinositol 3-kinase (PI3K) and protein kinase B (Akt). This pathway plays an important role in cell growth, proliferation, metabolism, and other processes [51,52,53] and also plays a key role in inflammatory responses. Mechanistic studies have demonstrated that PI3K-Akt signaling activates downstream transcriptional regulators, particularly NF-κB and mTOR, thereby stimulating the expression of key pro-inflammatory factors such as TNF-α, IL-1β, and IL-6, ultimately amplifying inflammatory cascades [54,55,56]. Although research on the functional role and mechanistic involvement of the PI3K-Akt signaling pathway in PRRSV pathogenesis remain limited, some studies suggests its significant contribution to PRRSV infection, such as PRRSV, can promote the production of IL-17 by activating the PI3K p38MAPK signaling pathway and promote pulmonary inflammation development [57]; baicalein-mediated inhibition of the EGFR-PI3K-Akt cascade effectively impedes PRRSV entry while enhancing the host immune response to PRRSV infection [58]. Based on a comprehensive analysis of our experimental data, we propose that the PI3K-Akt-NF-κB signaling cascade represents a crucial pathway through which PLE modulates PRRSV-induced inflammation. Validation experiments conducted in PAMs demonstrated that PRRSV infection potently activates the PI3K-Akt-NF-κB signaling pathway, resulting in elevated inflammation. In contrast, treatment with BNPP effectively suppresses the activation of this pathway, particularly through inhibition of Akt and p65 phosphorylation, consequently attenuating inflammatory levels.

Notably, 2-AG directly inhibits PRRSV-induced activation of the PI3K-Akt-NF-κB pathway. 2-AG exhibits an effect comparable to BNPP, suggesting that BNPP—as the inhibitor of PLE—elevates 2-AG concentration by blocking its PLE-mediated degradation. Consequently, this suppression of 2-AG catabolism leads to inhibition of the PI3K-Akt-NF-κB signaling pathway and subsequent downregulation of inflammatory responses. This mechanism likely underlies the observed reduction in PRRSV-induced inflammation following PLE inhibition. While our findings demonstrate that 2-AG suppresses PRRSV-triggered inflammation, the precise molecular mechanisms remain unclear and warrant further investigation. Elucidating this mechanism will provide critical insights into the role of 2-AG in modulating PRRSV infection, thereby facilitating the development of novel 2-AG-based strategies for disease prevention and control. Moreover, our study reveals that pharmacological regulation of PLE-mediated endocannabinoid hydrolysis can restore inflammatory homeostasis during PRRSV infection, offering novel intervention opportunities for both viral and inflammatory diseases.

## 5. Conclusions

**Figure 8:**
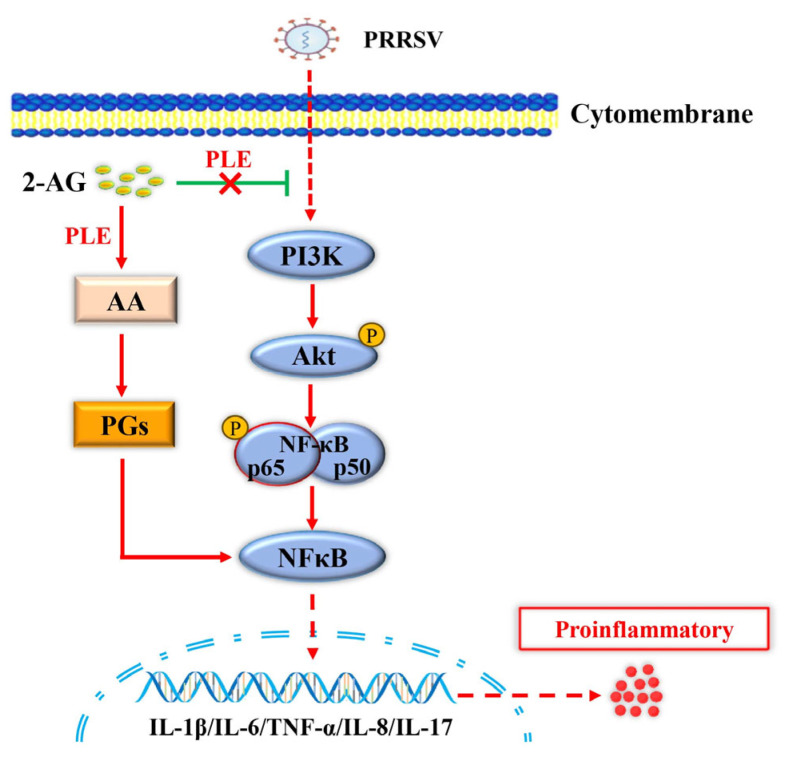
PLE-mediated hydrolysis of endocannabinoids exacerbates PRRSV-induced inflammation via of the PI3K-Akt-NF-κB signaling pathway. During PRRSV infection, PLE hydrolyzes 2-AG to produce pro-inflammatory mediators (AA and PGs), directly exacerbating inflammation. Concurrently, this hydrolysis diminishes 2-AG’s inhibitory effect on PI3K-Akt-NF-κB pathway activation, further amplifying the inflammatory response.

In conclusion, this study reports the identification of PLE expression in PAMs for the first time, elucidates the role of PLE in inflammation caused by PRRSV infection, and reveals sectional mechanisms of action: on the one hand, due to the hydrolysis of PLE, the inhibition of PI3K-Akt-NF-κB activation by 2-AG during PRRSV infection is weakened, leading to an increase in inflammation; on the other hand, PLE hydrolyzes 2-AG to generate pro-inflammatory mediators such as AA and PGs, which can directly lead to an increase in inflammation after PRRSV infection (Figure 8). In summary, PLE exerts a pro-inflammatory effect during PRRSV infection by intervening in the endogenous cannabinoid system. These findings not only deepen our understanding of PRRSV pathogenesis, but also provide a theoretical basis for developing therapeutic strategies targeting PLE activity to regulate PRRSV-associated inflammation, as well as for genetic breeding approaches to enhance PRRS resistance. Furthermore, PLE as a novel endogenous cannabinoid-degrading enzyme suggests its potential as a broad-spectrum therapeutic target for various inflammation-related disorders beyond PRRSV infection.

## Data Availability

All data generated during this study are included in this article and its Supplementary Information Files. The names of the repository/repositories and accession number(s) can be found below: GEO accession: GSE296917.

## Supplementary Materials

The following supporting information can be downloaded at https://www.mdpi.com/article/10.3390/cells14161227/s1, Figure S1: Prokaryotic functional expression of PLE6 identified by SDS-PAGE; Figure S2: Identification of prokaryotic functional expression of PLE6 hydrolytic activity; Table S1: RT-PCR Amplification and Sequencing Primers; Table S2: RT-qPCR Primers; Table S3: siRNA sequences; Tables S4–S8: Amino acid sequence of positive clone mutation site.
