# Supplementary material for: Pig Liver Esterase Hydrolysis of 2-Arachidonoglycerol Exacerbates PRRSV-Induced Inflammation via PI3K-Akt-NF-κB Pathway
Source: Cells. 2025 Aug 8;14(16):1227. doi: 10.3390/cells14161227 (PMC12384694; doi:10.3390/cells14161227)
Supplement: Supplementary file 1 [file cells-14-01227-s001.zip › cells-3785507-supplementary.pdf]

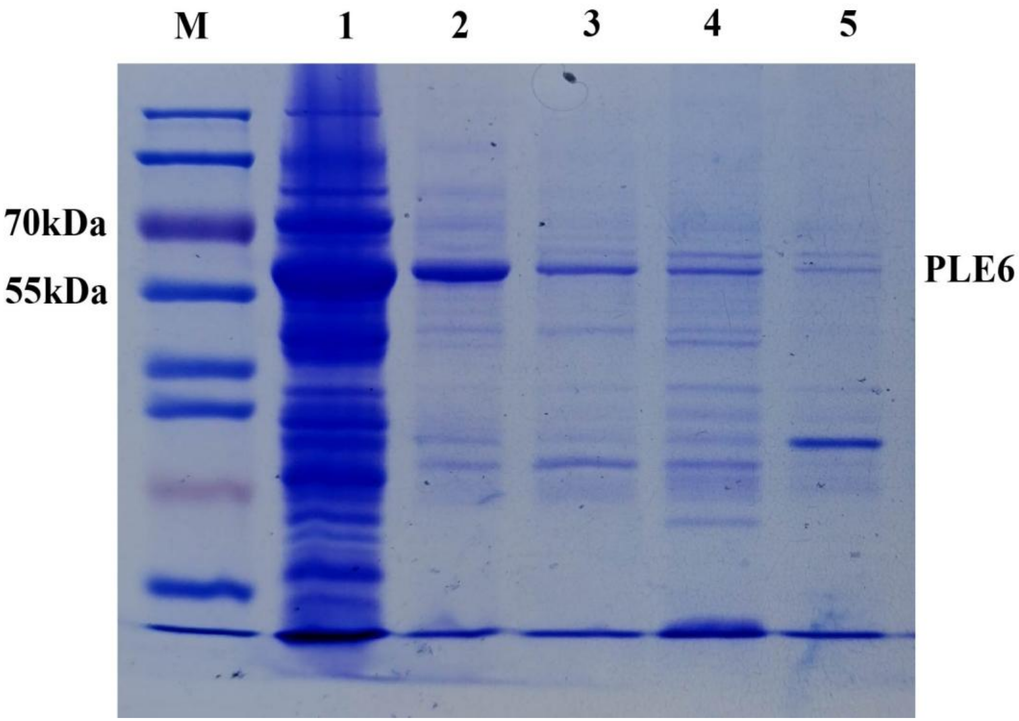

Supplementary Figure S1 Prokaryotic functional expression of PLE6 identified by SDS-PAGE. 1 Bacterial fragmentation supernatant 2 50mM imidazole elution 3 100mM imidazole elution 4 200mM imidazole elution 5 500mM imidazole elution

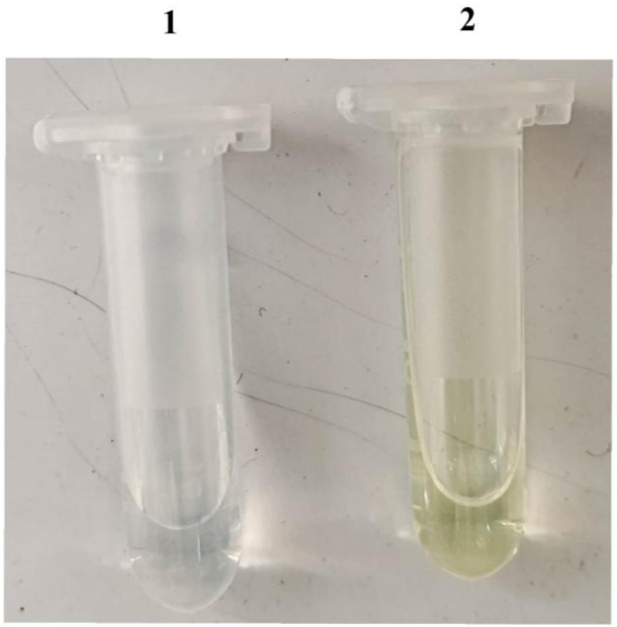

Supplementary Figure S2 Identification of prokaryotic functional expression of PLE6 hydrolytic activity. 1 Blank control 2 Bacterial fragmentation supernatant

Table S1 RT-PCR Amplification and Sequencing Primers

Primer	Sequence (5'-3')
PLE Forward	ATGTGGCTTCTCCCGCTGGTCCTGA
PLE Reverse	CCTCAAGGTCAGCCGAGCCTCCCCT
M13(-47)	CGCCAGGGTTTTCCCAGTCACGAC
M13(-48)	AGCGGATAACAATTTCACACAGGA
PLE-M	CCTGGGCATCTGGGGATTCTT

Table S2 RT-qPCR Primers

Primer	Sequence (5’-3’)
IL-1β Forward	GCTGGAGGATATAGACCCC
IL-1β Reverse	GTTGGGGTACAGGGCAGAC
IL-6 Forward	ACAAAGCCACCACCCCTAAC
IL-6 Reverse	CGTGGACGGCATCAATCTCA
IL-8 Forward	AGTTTTCCTGCTTTCTGCAGCT
IL-8 Reverse	TGGCATCGAAGTTCTGCACT
IL-17 Forward	CTCTCCAACGCAACGAGGAC
IL-17 Reverse	GTCACCATCACTTTCTCCAGCC
TNF-α Forward	TTCCAGCTGGCCCCTTGAGC
TNF-α Reverse	GAGGGCATTGGCATACCCAC
PI3K Forward	CGTGGTGCTGAGAGAGGACA
PI3K Reverse	CTGGCTGGTGGGCAAAA
Akt Forward	AGCTCATCCTCATGGAGGAG
Akt Reverse	CATACACATCCTGCCACACG
NF-κB Forward	GAATTCCAGTACTTGCCAGA
NF-κB Reverse	TCTCATAGGTCCTTTTGCG
GAPDH Forward	GAAGGTCGGAGTGAACGGAT
GAPDH Reverse	CATGGGTAGAATCATACTGGAACA

Table S3 siRNA sequences

Primer	Sequence (5'-3')
siPLE-1	CCACCUCCUACCCUCCCAUTT
	AUGGGAGGGUAGGAGGUGGTT
siPLE-2	CCACCACCUCGGCUGUCUUTT
	AAGACAGCCGAGGUGGUGGTT
si negative control	UUCUCCGAACGUGUCACGUTT
	ACGUGACACGUUCGGAGAATT

Table S4 Amino acid sequence of positive clone mutation site

**Remove the mutation sites of the 18 signal peptides at the N-terminus**	2	12	14	15	20	22	28	29	33	35	40	44
73	E	E	E	E	E	E	E	E	E	E	E	E
75	I	I	I	I	I	I	I	I	I	I	I	I
76	G	G	G	G	G	G	G	G	G	G	G	G
77	G	G	G	G	G	G	G	G	G	G	G	G
80	L	L	L	L	L	L	L	L	L	L	L	L
87	R	R	R	R	R	R	R	R	R	R	R	R
92	I	I	I	I	I	I	I	I	I	I	I	I
93	P	P	P	P	P	P	P	P	P	P	P	P
129	V	V	V	V	V	V	V	V	V	V	V	V
133	S	S	S	S	S	S	S	S	S	S	S	S
134	T	T	T	T	T	T	T	T	T	T	T	T
138	L	L	L	L	L	L	L	L	L	L	L	L
139	A	A	A	A	A	A	A	A	A	A	A	A
234	F	F	F	F	F	F	F	F	F	F	F	F
236	A	A	A	A	A	A	A	A	A	A	A	A
237	G	G	G	G	G	G	G	G	G	G	G	G
285	F	F	F	F	F	F	F	F	F	F	F	F
286	F	F	F	L	F	F	F	F	F	F	F	F
287	A	A	A	T	A	A	A	A	A	A	A	A
290	L	L	L	L	L	L	L	L	L	L	L	L
294	P	P	P	Q	P	P	P	P	P	P	P	P
302	T	T	T	T	T	T	T	T	T	T	T	T
459	A	A	A	F	A	A	A	A	A	A	A	A
461	F	F	F	L	F	F	F	F	F	F	F	F
463	R	R	R	K	R	R	R	R	R	R	R	R
**Corresponding subtype**	PLE6	PLE6	PLE6	undetermined	PLE6	PLE6	PLE6	PLE6	PLE6	PLE6	PLE6	PLE6

Table S5 Amino acid sequence of positive clone mutation site

**Remove the mutation sites of the 18 signal peptides at the N-terminus**	50	52	53	56	58	62	63	65	66	68	70	84	91
4	E	E	E	E	E	E	E	E	E	E	E	E	E
75	I	I	I	I	I	I	I	I	I	I	I	I	I
76	G	G	G	G	G	G	G	G	G	G	G	G	G
77	G	G	G	G	G	G	G	G	G	G	G	G	G
80	L	L	L	L	L	L	L	L	L	L	L	L	L
87	R	R	R	R	R	R	R	R	R	R	R	R	R
92	I	I	I	I	I	I	I	I	I	I	I	I	I
93	P	P	P	P	P	P	P	P	P	P	P	P	P
129	V	V	V	V	V	V	V	V	V	V	V	V	V
133	S	S	S	S	S	S	S	S	S	S	S	S	S
134	T	T	T	T	T	T	T	T	T	T	T	T	T
138	L	L	L	L	L	L	L	L	L	L	L	L	L
139	A	A	A	A	A	A	A	A	A	A	A	A	A
234	F	F	F	F	F	F	F	F	F	F	F	F	F
236	A	A	A	A	A	A	A	A	A	A	A	A	A
237	G	G	G	G	G	G	G	G	G	G	G	G	G
285	F	F	F	F	F	F	F	F	F	F	F	F	F
286	F	F	F	F	F	F	F	F	F	F	F	F	F
287	A	A	A	A	A	A	A	A	A	A	A	A	A
290	L	L	L	L	L	L	L	L	L	L	L	L	L
294	P	P	P	P	P	P	P	P	P	P	P	P	P
302	T	T	T	T	T	T	T	T	T	T	T	T	T
459	A	A	A	A	A	A	A	A	A	A	A	A	A
461	F	F	F	F	F	F	F	F	F	F	F	F	F
463	R	R	R	R	R	R	R	R	R	R	R	R	R
**Corresponding subtype**	PLE6	PLE6	PLE6	PLE6	PLE6	PLE6	PLE6	PLE6	PLE6	PLE6	PLE6	PLE6	PLE6

Table S6 Amino acid sequence of positive clone mutation site

**Remove the mutation sites of the 18 signal peptides at the N-terminus**	3	8	15	28	37	45	50	52	64	67	68	75	76	79	80
73	E	E	E	E	E	E	D	E	E	E	D	D	E	E	E
75	I	I	I	I	I	I	V	I	I	I	V	V	I	I	I
76	G	G	G	G	G	G	A	G	G	G	A	A	G	G	G
77	G	G	G	G	G	G	G	G	G	G	G	G	G	G	G
80	L	L	L	L	L	L	T	L	L	L	T	T	L	L	L
87	R	R	R	R	R	R	G	R	R	R	G	G	R	R	R
92	I	I	I	I	I	I	I	I	I	I	I	I	I	I	I
93	P	P	P	P	P	P	P	P	P	P	P	P	P	P	P
129	V	V	V	V	V	V	V	V	V	V	V	V	V	V	V
133	S	S	S	S	S	S	S	S	S	S	S	S	S	S	S
134	T	T	T	T	T	T	T	T	T	T	T	T	T	T	T
138	L	L	L	L	L	L	L	L	L	L	L	L	L	L	L
139	A	A	A	A	A	A	A	A	A	A	A	A	A	A	A
234	F	F	F	F	F	L	L	F	L	F	L	F	F	F	F
236	A	A	A	A	A	A	A	A	A	A	A	A	A	A	A
237	G	G	G	G	G	G	G	G	G	G	G	G	G	G	G
285	F	F	F	F	F	F	F	F	F	F	F	F	F	F	F
286	F	F	F	F	F	L	L	F	L	F	L	F	F	F	F
287	A	A	A	A	A	T	T	A	T	A	T	A	A	A	A
290	L	L	L	L	L	L	L	L	L	L	L	L	L	L	L
294	P	P	P	P	P	Q	Q	P	Q	P	Q	P	P	P	P
302	T	T	T	T	T	T	T	T	T	T	T	T	T	T	T
459	A	A	A	A	A	F	F	A	A	A	A	A	A	A	A
461	F	F	F	F	F	L	L	F	F	F	F	F	F	F	F
463	R	R	R	R	R	K	K	R	R	R	R	R	R	R	R
**Corresponding subtype**	PLE6	PLE6	PLE6	PLE6	PLE6	PLED5	PLEG5	PLE6	undetermined	PLE6	PLEG9	PLEG14	PLE6	PLE6	PLE6

Table S7 Amino acid sequence of positive clone mutation site

Remove the mutation sites of the 18 signal peptides at the N-terminus	5	6	7	8	10	13	14	15	18	20	21	22	23	26	27	28	29	32	34	35	38	40
4	E	E	E	E	E	E	E	E	E	E	E	E	E	E	E	E	E	E	E	E	E	E
75	I	I	I	I	I	I	I	I	I	I	I	I	I	I	I	I	I	I	I	I	I	I
76	G	G	G	G	G	G	G	G	G	G	G	G	G	G	G	G	G	G	G	G	G	G
77	G	G	G	G	G	G	G	G	G	G	G	G	G	G	G	G	G	G	G	G	G	G
80	L	L	L	L	L	L	L	L	L	L	L	L	L	L	L	L	L	L	L	L	L	L
87	R	R	R	R	R	R	R	R	R	R	R	R	R	R	R	R	R	R	R	R	R	R
92	I	I	I	I	I	I	I	I	I	I	I	I	I	I	I	I	I	I	I	I	I	I
93	P	P	P	P	P	P	P	P	P	P	P	P	P	P	P	P	P	P	P	P	P	P
129	V	V	V	V	V	V	V	V	V	V	V	V	V	V	V	V	V	V	V	V	V	V
133	S	S	S	S	S	S	S	S	S	S	S	S	S	S	S	S	S	S	S	S	S	S
134	T	T	T	T	T	T	T	T	T	T	T	T	T	T	T	T	T	T	T	T	T	T
138	L	L	L	L	L	L	L	L	L	L	L	L	L	L	L	L	L	L	L	L	L	L
139	A	A	A	A	A	A	A	A	A	A	A	A	A	A	A	A	A	A	A	A	A	A
234	F	F	F	F	F	F	F	F	F	F	F	F	F	F	F	F	F	F	F	F	F	F
236	A	A	A	A	A	A	A	A	A	A	A	A	A	A	A	A	A	A	A	A	A	A
237	G	G	G	G	G	G	G	G	G	G	G	G	G	G	G	G	G	G	G	G	G	G
285	F	F	F	F	F	F	F	F	F	F	F	F	F	F	F	F	F	F	F	F	F	F
286	F	F	F	F	F	F	F	F	F	F	F	F	F	F	F	F	F	F	F	F	F	F
287	A	A	A	A	A	A	A	A	A	A	A	A	A	A	A	A	A	A	A	A	A	A
290	L	L	L	L	L	L	L	L	L	L	L	L	L	L	L	L	L	L	L	L	L	L
294	P	P	P	P	P	P	P	P	P	P	P	P	P	P	P	P	P	P	P	P	P	P
302	T	T	T	T	T	T	T	T	T	T	T	T	T	T	T	T	T	T	T	T	T	T
459	A	A	A	A	A	A	A	A	A	A	A	A	A	A	A	A	A	A	A	A	A	A
461	F	F	F	F	F	F	F	F	F	F	F	F	F	F	F	F	F	F	F	F	F	F
463	R	R	R	R	R	R	R	R	R	R	R	R	R	R	R	R	R	R	R	R	R	R
Corresponding subtype	PLE6	PLE6	PLE6	PLE6	PLE6	PLE6	PLE6	PLE6	PLE6	PLE6	PLE6	PLE6	PLE6	PLE6	PLE6	PLE6	PLE6	PLE6	PLE6	PLE6	PLE6	PLE6

Table S8 Amino acid sequence of positive clone mutation site

**Remove the mutation sites of the 18 signal peptides at the N-terminus**	1	2	3	7	9	11	15	16	20	32	35	38	39
4	E	E	E	E	E	E	E	E	E	E	E	E	E
75	I	I	I	I	I	I	I	I	I	I	I	I	I
76	G	G	G	G	G	G	G	G	G	G	G	G	G
77	G	G	G	G	G	G	G	G	G	G	G	G	G
80	L	L	L	L	L	L	L	L	L	L	L	L	L
87	R	R	R	R	R	R	R	R	R	R	R	R	R
92	I	I	I	I	I	I	I	I	I	I	I	I	I
93	P	P	P	P	P	P	P	P	P	P	P	P	P
129	V	V	V	V	V	V	V	V	V	V	V	V	V
133	S	S	S	S	S	S	S	S	S	S	S	S	S
134	T	T	T	T	T	T	T	T	T	T	T	T	T
138	L	L	L	L	L	L	L	L	L	L	L	L	L
139	A	A	A	A	A	A	A	A	A	A	A	A	A
234	F	F	F	F	F	F	F	F	F	F	F	F	F
236	A	A	A	A	A	A	A	A	A	A	A	A	A
237	G	G	G	G	G	G	G	G	G	G	G	G	G
285	F	F	F	F	F	F	F	F	F	F	F	F	F
286	F	F	F	F	F	F	F	F	F	F	F	F	F
287	A	A	A	A	A	A	A	A	A	A	A	A	A
290	L	L	L	L	L	L	L	L	L	L	L	L	L
294	P	P	P	P	P	P	P	P	P	P	P	P	P
302	T	T	T	T	T	T	T	T	T	T	T	T	T
459	A	A	A	A	A	A	A	A	A	A	A	A	A
461	F	F	F	F	F	F	F	F	F	F	F	F	F
463	R	R	R	R	R	R	R	R	R	R	R	R	R
**Corresponding subtype**	PLE6	PLE6	PLE6	PLE6	PLE6	PLE6	PLE6	PLE6	PLE6	PLE6	PLE6	PLE6	PLE6
